# Quality of prenatal care in riverside communities of the Brazilian Amazon

**DOI:** 10.1590/0034-7167-2025-0204

**Published:** 2026-11-06

**Authors:** Edson Silva do Nascimento, Anderson Lima Cordeiro da Silva, Bibiane Dias Miranda Parreira, Mônica Maria de Jesus Silva, Nayara Gonçalves Barbosa, Alexandre Faisal Cury, Flávia Gomes-Sponholz

**Affiliations:** I Universidade de São Paulo. Ribeirão Preto, São Paulo, Brazil; II Universidade Federal do Triângulo Mineiro. Uberaba, Minas Gerais, Brazil; III Universidade de São Paulo. São Paulo, São Paulo, Brazil

**Keywords:** Nursing, Quality of Health Care, Primary Health Care, Prenatal Care, Rural Health Services., Enfermería, Calidad de la Atención de Salud, Atención Primaria de Salud, Atención Prenatal, Servicios de Salud Rural.

## Abstract

**Objectives:**

to describe the quality of prenatal care offered to pregnant women living in riverside communities of Manaus, Amazonas.

**Methods:**

cross-sectional study conducted in December 2022 in a Fluvial Primary Health Care Unit. Twenty-five pregnant women (≥18 years), residents in the coverage area and with at least one prenatal visit, participated. Data were collected through interviews and medical records review and analyzed using descriptive statistics.

**Results:**

all participants received prenatal care, but half did not complete six consultations. Laboratory tests, vaccines, and clinical-obstetric procedures were offered, with blood pressure measurement and fetal auscultation most consistently performed. Gaps were identified in counseling on warning signs and referral maternity services, although most women reported satisfaction.

**Conclusions:**

despite expanded access provided by the fluvial unit, challenges remain in ensuring continuity and equity of care in remote areas, indicating the need for improvements.

## RESUMEN

**Objetivos:**

describir la calidad de la atención prenatal ofrecida a gestantes de comunidades ribereñas de Manaos, Amazonas.

**Métodos:**

estudio transversal realizado en diciembre de 2022 en una Unidad Básica de Salud Fluvial. Participaron 25 gestantes (≥18 años), residentes en el área de cobertura y con al menos una consulta prenatal registrada. Los datos se recolectaron mediante entrevistas y revisión de registros y se analizaron con estadística descriptiva.

**Resultados:**

todas recibieron atención prenatal, pero la mitad no completó seis consultas. Se ofrecieron exámenes, vacunas y procedimientos clínico-obstétricos, destacándose la medición de la presión arterial y la auscultación fetal. Se identificaron brechas en las orientaciones sobre señales de riesgo y en la referencia a maternidades, aunque la mayoría refirió satisfacción.

**Conclusiones:**

a pesar de la ampliación del acceso promovida por la unidad fluvial, persisten desafíos para garantizar continuidad y equidad del cuidado en áreas remotas, lo que indica la necesidad de mejoras.

## INTRODUCTION

Prenatal care is considered by the Ministry of Health a decisive component of the maternal child care continuum, as it contributes to safer pregnancy, childbirth, and postpartum experiences, promoting the well being of women and newborns^(1)^. This follow up enables the early identification of risk situations and strengthens the bond between users and primary care teams^(2)^. With the implementation of the Prenatal and Birth Humanization Program (2000) and the Alyne Network (2024), both initiatives of the Ministry of Health, efforts have been made to improve the structure, organization, and expansion of maternal child care, broadening the focus on equity and reproductive rights while maintaining prenatal care as a central axis of the care network^(3)^.

The Brazilian context is characterized by broad territorial, ethnic, and cultural diversity^(4)^. Within this landscape of regional inequalities, the Brazilian Amazon stands out as a territory where structural, logistical, and organizational limitations of health services are expressed more intensely, making it an emblematic context for analyzing equity in prenatal care. Among the population groups living under challenging conditions of access to health services, Amazonian riverside communities are notable; their ways of life are organized along riverbanks and depend on fluvial logistics^(4)^. In these localities, geographic distances, the scarcity of regular transportation, and the structural limitations of public services constitute significant barriers to continuous and high quality prenatal follow up^(5,6)^. Understanding the living and health conditions of these pregnant women is essential for guiding culturally sensitive care actions and strategies to expand access^(6,7)^.

These structural and territorial inequalities are reflected concretely in prenatal care indicators across different contexts of the Amazon, highlighting how access limitations affect populations living in remote areas. Although referring specifically to Indigenous populations, data from the Special Indigenous Health District of Manaus (“*DSEI/Manaus*”) provide important insights into the structural constraints of prenatal care in remote Amazonian territories, which also affect other traditional populations, such as riverside communities. Recent *DSEI/Manaus* reports indicate that approximately 52% of Indigenous pregnant women completed prenatal care with six or more consultations^(8)^. Among riverside pregnant women specifically, a study conducted in communities of the middle Solimões river region with 28 women showed a high proportion of first pregnancies during adolescence and a high frequency of home births, reflecting reproductive patterns characteristic of the local context^(9)^. These findings reinforce the importance of investigating not only coverage but also the quality of prenatal care among Amazonian riverside populations.

Beyond the description of coverage indicators, recent studies highlight that the organization of health services and territorial conditions play a central role in shaping prenatal care in Amazonian communities^(5,6)^. The territorial heterogeneity of the Amazon - characterized by vast distances, dispersed populations, and dependence on fluvial logistics - directly affects the organization and functioning of health services. In this context, the periodic availability of fluvial health units, operational limitations, and the need to adapt care flows condition the provision of consultations, examinations, and counseling, requiring that SUS care models be structured according to the socioeconomic and epidemiological specificities of each locality^(10)^.

In this regard, the National Primary Care Policy sought to expand service provision and reorganize care to meet the specific needs of populations living in hard to reach areas. As part of this strategy, the Ministry of Health established, through Ordinance No. 2,191 of August 3, 2010, the Fluvial and Riverside Family Health Teams, focusing on care for communities in the Legal Amazon and the Pantanal of Mato Grosso do Sul^(11,12)^.

Recent evidence indicates high prenatal care coverage in Brazil, with estimates of approximately 98.7% in small and medium sized municipalities^(13)^. However, this broad coverage does not translate uniformly into timely initiation of care, an adequate number of consultations, or the systematic performance of recommended procedures, with important differences persisting across regions and care contexts^(14)^. In hard to reach areas, such as certain localities in the Amazon, these asymmetries reveal the limits of conventional models of primary care organization, reinforcing the need for care strategies adapted to territorial and population specific characteristics^(10)^.

This situation is not limited to Brazil. In other countries of the Global South, such as the region of Assam, India, women report limited knowledge of reproductive rights and face difficulties accessing family planning services^(15)^. This parallel broadens the understanding that the barriers faced by traditional populations in remote contexts stem from shared structural and territorial determinants, rather than being an exclusive feature of the Amazonian or Brazilian context. In this sense, similar obstacles - related to geographic, cultural, and economic barriers - have been described as factors shaping reproductive practices and limiting women’s autonomy in different settings^(16)^.

This investigation is justified by the need to understand how geographic and socioeconomic conditions influence access to and continuity of prenatal care in remote regions of the Amazon. These factors remain underexplored in the literature and constitute central elements for improving equity and the quality of obstetric care offered to riverside women.

### Study relevance

This article is part of the Master’s Dissertation titled “Analysis of the quality of prenatal care in riverside communities of the Brazilian Amazon,” defended at the University of São Paulo^(17)^.

## OBJECTIVES

To describe the quality of prenatal care offered to riverside pregnant women in the municipality of Manaus, Amazonas.

## METHODS

### Ethical considerations

The study was approved by the Research Ethics Committee the of *Escola de Enfermagem de Ribeirão Preto da Universidade de São Paulo* (Ribeirão Preto School of Nursing at the University of São Paulo), in accordance with Resolution No. 466/2012 of the National Health Council, which establishes guidelines for research involving human subjects.

### Setting, period and location of study

This is a descriptive study conducted between December 13 and 21, 2022, in a Fluvial Primary Health Care Unit (“*UBSF*”) that travels along the Amazon River channel, a rural area in the municipality of Manaus, Amazonas. The study design followed the recommendations of the Strengthening the Reporting of Observational Studies in Epidemiology (STROBE).

The Amazon River channel is covered by the Family Health Strategy through a *UBSF* linked to the Municipal Health Secretariat, which itinerantly provides monthly care to communities dispersed along the fluvial route. Across the region, there are six hubs of Riverside Primary Health Care Units, encompassing 23 localities - among communities, streams, lakes, and river branches - served by the *UBSF* and by Community Health Workers who remain in these areas even when the vessel is not present^(18)^.

The region had approximately 2,333 inhabitants^(18)^ distributed along the river course, in an extensive aquatic area far from the administrative center of Manaus. Subsistence activities include family farming (notably the production and sale of manioc flour), artisanal fishing, agricultural extractivism, and, occasionally, hunting.

### Population or sample, inclusion and exclusion criteria

A total of 25 pregnant women followed by the *UBSF* during prenatal care participated in the study, selected through convenience sampling due to the impossibility of prior access to information that would allow sample size calculation, given the logistical constraints of a fluvial expedition. Inclusion occurred according to the established criteria and the presence of the pregnant women at the prenatal consultations conducted during the *UBSF’s* stay in the communities. It is acknowledged that the use of convenience sampling may introduce selection bias and limit the representativeness of the results. However, this strategy proved feasible in light of the region’s geographic barriers and allowed for a consistent description of the reality of the women receiving care.

Inclusion criteria were being 18 years of age or older, residing in a riverside community within the *UBSF*’s coverage area, and having at least one recorded prenatal consultation. Exclusion criteria included pregnant women who discontinued follow up after the first consultation, those who did not understand the Portuguese language, or those with total or partial hearing impairment that would prevent the interview. During data collection, no pregnant woman was excluded, either due to discontinuation of follow up or sensory limitations. Data collection took place in the context of a fluvial expedition, which made active case finding unfeasible, restricting participation to pregnant women who were attended in person.

### Study proctocol

Before data collection began, a preparatory meeting was held at the Rural Health District, during which the researcher and the project were presented to the *UBSF* team. The meeting also included the participation of the vessel operators, with the purpose of understanding the functioning of the unit and organizing the flow of care for the population, ensuring a welcoming reception for the study participants.

Data collection took place on the vessel itself, after the prenatal consultation, from Monday to Sunday, between 8:00 a.m. and 12:00 p.m. and from 2:00 p.m. to 5:00 p.m. The interviews were conducted by the lead researcher, who explained the study’s objectives, procedures, and potential risks. After reading and signing the Informed Consent Form, the interviews were carried out individually and privately, using a printed instrument. There were no refusals or missing data.

The variables used to characterize the sociodemographic and economic profile included age, race/skin color, marital status, education level, religion, civil status, occupation, individual and household income, family provider, and number of household residents.

The assessment of prenatal care quality included completion of the most recent prenatal care, clinical obstetric procedures, preventive measures, health counseling received, and satisfaction with the follow up.

The sociodemographic characterization instrument was developed based on national and international scientific literature on populations living in vulnerable contexts.

The instrument used to assess the quality of care was adapted from a study conducted in Nampula, Mozambique^(19)^, selected due to its comprehensiveness and applicability in contexts of vulnerability and limited access, such as Amazonian riverside communities, allowing for direct measurement of the quality of care received.

### Analysis of results and statistics

The data were double entered into an electronic spreadsheet in Microsoft Excel for consistency checking and analyzed using IBM SPSS Statistics, version 20.0, employing descriptive statistics.

Continuous variables were presented as means and standard deviations, and categorical variables as absolute and relative frequencies (n, %).

It is important to note that, due to the cross sectional design and the small convenience sample, it was not possible to establish causal relationships. The access barriers typical of the fluvial context represent additional limitations for the generalizability of the study.

## RESULTS

The study included 25 pregnant women living in communities along the Amazon River channel, who were followed during prenatal care at a Fluvial Primary Health Unit (UBSF). Most were young (64% between 20 and 29 years old), lived in stable unions (88%), had low levels of schooling, and were in socially and economically vulnerable conditions, relying on income transfer programs such as Bolsa Família (68%), in addition to being linked to local agricultural activities (Table 1). This profile reveals the overlap between social vulnerabilities and structural limitations, especially regarding access and income, which shape the care trajectories of riverside pregnant women.

**Table 1:** Socioeconomic characteristics of pregnant women living along the Amazon River channel receiving prenatal care at the Fluvial Primary Health Unit, Manaus, Amazonas, Brazil, 2023

Variables	n	%
**Age group (full years)**		
18 -| 19	4	16
20 -| 29	16	64
30 -| 39	4	16
40 -| 49	1	4
**Self-reported race/ethnicity**		
Brown	21	84
Yellow	3	12
Black	1	4
**Formal education**		
Incomplete Elementary School	7	28
Complete High School	7	28
Complete Elementary School	5	20
Incomplete High School	3	12
Complete Higher Education	2	8
Incomplete Higher Education	1	4
**Religion**		
Catholic	10	40
Evangelical	7	28
Believes in God but has no religion	2	8
Other	6	24
**Marital Status**		
Married	22	88
Single	3	12
**Occupation**		
Homemaker	19	76
Formal labor market	2	8
Unemployed	2	8
Informal labor market	1	4
Student	1	4
**Income Source**		
Bolsa Família	17	68
No income	3	12
Formal labor market	2	8
State assistance	1	4
Informal labor market	1	4
Pension	1	4
**Individual income (minimum wages)**		
≤ 1	22	88
1-| 2	1	4
No individual income	2	8
**Household income (minimum wages)**		
≤ 1	13	52
1-| 2	11	44
No household income	1	4
**Family provider**		
Partner	8	32
Both (participant and partner)	10	40
Participant	3	12
Parents	3	12
Others	1	4
**Number of people livinig in the household**		
2	4	16
3	8	32
4	5	20
More than 5	8	32
**Total**	25	100.0

All participants attended prenatal care. Approximately half did not complete six consultations, mainly due to transportation and mobility difficulties characteristic of the riverside context, while the other half reached the minimum recommended number, supported by family assistance, the availability of transportation, and closer proximity to the *UBSF* (Table 2).

**Table 2:** Characteristics of the most recent prenatal care received by pregnant women living along the Amazon River channel and followed at Fluvial Primary Health Unit, Manaus, Amazonas, Brazil, 2023

Variables	Sim		Não	
	n	%	n	%
**GA at initiation of prenatal care (in weeks)**				
1 -| 4			9	36
5 -| 8			10	40
9 -| 12			6	24
**Reasons for attending < 6 appointments**				
Difficulties related to work/school	3	12	22	88
Did not know she was pregnant	3	12	22	88
Did not accept the pregnancy	3	12	22	88
Thought her belly looked small	2	8	23	92
Had no one to accompany her	2	8	22	92
Long waiting time for care	1	4	24	96
Did not think it was important to start prenatal care early	1	4	24	96
Had no one to leave her children with	1	4	24	96
The Fluvial Primary Health Unit (UBSF) is far away	1	4	24	96
**Reasons for attending > 6 appointments**				
Family and partner support	19	76	6	24
Recognizes the importance of prenatal care	19	76	6	24
Fluvial Primary Health Unit (UBSF) located near home	19	76	6	24
Access to transportation	18	72	7	28
Desire to take better care of the pregnancy	18	72	7	28
Health professionals provide good care	16	76	6	24
**Total number of prenatal consultations completed (up to the day of the interview)**				
1-| 3			6	24
4-| 5			6	24
≥ 6			13	52
**Total**			**25**	**100.0**

There was a consistent provision of basic examinations, including ultrasound (88%), as well as vaccines and clinical obstetric procedures, with emphasis on blood pressure measurement (60%) and fetal auscultation (60%). Vaccination coverage showed satisfactory levels for hepatitis B (84%), tetanus (80%), and influenza (88%), although adherence to the COVID 19 vaccine was lower (48%), considering the three doses recommended by the Ministry of Health (Table 3). These findings highlight the role of the Fluvial Primary Health Unit (UBSF) as a mobile care strategy capable of ensuring the delivery of essential services even in hard to reach regions.

**Table 3:** Characteristics of pregnant women living along the Amazon River channel and receiving prenatal care at the Fluvial Primary Health Unit according to the performance of clinical obstetric procedures and preventive measures during prenatal care, Manaus, Amazonas, Brazil, 2023

Variables	None		1		2		3		≥4	
	n	%	n	%	n	%	n	%	n	%
Number of blood pressure assessments	2	8	2	8	1	4	5	20	15	60
Number of weight assessments	2	8	1	4	2	8	6	24	14	56
Number of iron sulfate supplements offered	-	-	7	28	5	20	4	16	9	36
Number of uterine height measurements	1	4	2	8	1	4	7	28	12	48
Number of fetal heart sound (FHS) auscultations	2	8	1	4	2	8	3	12	15	60
Hepatitis B vaccine	1	4	3	12	-	-	21	84	-	-
Tdap vaccine	5	20	20	80	-	-	-	-	-	-
Tetanus vaccine	1	4	1	16	-	-	20	80	-	-
Influenza vaccine	3	12	22	88	-	-	-	-	-	-
COVID-19 vaccine	1	4	1	4	7	28	12	48	4	16

Records related to signs of gestational risk were infrequent (8-16%), indicating a gap in the quality of prenatal care provided to pregnant women.

The most frequent counseling topics addressed healthy eating (88%), postpartum care (76%), and reproductive planning (72%), while warning signs of gestational risk were rarely discussed. This gap reflects the impact of time constraints and staff turnover during fluvial expeditions.

Satisfaction with the care received was predominant (96%), with emphasis on the team’s cordiality and the value attributed to the presence of the Fluvial Primary Health Unit (UBSF) in the communities. This positive perception highlights how the regular provision of services through fluvial expeditions helps reduce geographic barriers and strengthen the bond between the community and the health service.

## DISCUSSION

The group of pregnant women investigated was predominantly young, with low educational attainment and living in contexts of socioeconomic vulnerability. This condition is reflected both in their means of subsistence and in their difficulties accessing health services, aligning with findings from the national study on the adequacy of prenatal care^(1)^ and with results observed in Amazonian communities such as Vila Alvarães^(20)^. In this context, the National Policy for Comprehensive Health of Rural, Forest, and Water Populations plays a strategic role in expanding access to prenatal care among rural and traditional populations^(21,22)^.

The participants’ income came mostly from cash transfer programs, especially social assistance, which was often used to cover basic needs. Financial insufficiency is a recurrent issue in Amazonian riverside communities^(20)^ and, at certain times, was offset by subsistence agriculture and the local sale of products such as manioc^(20)^. These economic conditions restrict mobility and continuity of prenatal follow up, reinforcing the importance of social protection policies as a strategy to promote equity in access to health care.

Socioeconomic inequalities across Brazilian regions influence reproductive behavior, especially in contexts of greater vulnerability, where early pregnancy and multiparity are more frequently observed. This situation is exacerbated by geographic barriers and limited access to specialized services^(23)^. In the North and Northeast regions, late initiation and lower prenatal care coverage among women with low educational attainment are more closely associated with territorial and structural barriers than with lack of information^(23)^, highlighting the need for strategies that consider local specificities and ensure timely initiation of care.

The quality of prenatal care is linked to early initiation of follow up - preferably between the first and third months of pregnancy - along with adequate consultations, examinations, and consistent guidance regarding the referral maternity unit, all of which are essential factors for improving perinatal outcomes^(24)^.

Studies conducted in different regions of Brazil, including the North, Northeast, Southeast, and South, indicate that, overall, the average number of consultations and the timing of prenatal care initiation fall within the recommended period^(24,25)^. These results demonstrate the capacity of primary care to ensure adequate services, even in remote areas. Similarly, an investigation carried out in the Amazon region identified a high proportion of early initiation of prenatal care^(5)^, whereas in Altamira, Pará, late initiation and fewer consultations were observed^(26)^, revealing differences among Amazonian states in relation to ministerial recommendations.

In international contexts with dispersed populations and limited resources, a diverse panorama is observed, such as the high coverage in Vanuatu, an island nation in the South Pacific^(27)^, and the low frequency of consultations in rural areas of Zambia^(16)^.

In the region studied, prenatal care is provided monthly by a multidisciplinary team composed of physicians and nurses, an organization that does not follow the alternation of appointments recommended in national protocols^(3)^. This care configuration resembles what has been observed in rural areas of Manaus (AM) and Lagarto (SE)^(28)^, where limited staffing and long distances shape the care model. In the North and Northeast regions, prenatal care is often conducted primarily by nurses due to the shortage of physicians in inland municipalities^(29)^. In similar international settings, such as the Torres Strait Islands in Australia, nurse led care models have proven effective in ensuring timely initiation of prenatal care and increasing the average number of consultations^(30)^.

High adherence to laboratory tests at the first prenatal visit was observed, indicating compliance with basic screening protocols. The *UBSF* team expanded this routine by requesting additional tests, including serologies and malaria screening, tailoring care to the endemic conditions of the Amazon region. This performance contrasts with findings from other urban or non remote regions of the country, where the completion of these tests tends to be less frequent^(31)^.

Although malaria is endemic in the region, none of the pregnant women reported a positive diagnosis during follow up, according to the thick blood smear test, a procedure recommended by the *Rede Cegonha* strategy for prenatal care in the states of the Legal Amazon. Studies conducted in Manaus, Coari, and Colombia indicate greater vulnerability of pregnant women to infection. In Coari, the risk was found to be up to five times higher than that observed among non pregnant women^(32)^. These findings reinforce the need to maintain active surveillance and systematic malaria screening during prenatal care in order to reduce maternal and fetal risks.

Most pregnant women underwent the main clinical and preventive prenatal procedures, in accordance with Ministry of Health guidelines^(2,4)^. These results are consistent with studies carried out in remote areas of Northern Brazil, which highlight the importance of continuity of gestational care for the early detection of risks and complications^(22,31)^.

In the present study, blood pressure, weight, and height measurements were frequently performed by Community Health Workers as a strategy to optimize consultation time during the *UBSF’s* stay in the communities^(33)^. Vaccination coverage showed satisfactory indicators, higher than those reported in a study conducted in a maternity hospital in the Northeast region^(34)^. In contrast, research conducted in Nampula, Mozambique, showed lower coverage^(19)^ than that observed in Brazil^(30)^, highlighting disparities between remote contexts and the need to expand national vaccination coverage even with the free access guaranteed by the *Sistema Único de Saúde* (SUS, Brazilian Unified Health System).

Weaknesses and gaps were identified in prenatal educational activities, particularly regarding the promotion of vaginal birth, as also described in studies conducted in Brazil^(13)^ and Mozambique^(19)^. Team overload is recognized as a factor that limits the provision of counseling and compromises pregnant women’s preparation throughout the pregnancy puerperal cycle^(31)^. These shortcomings may delay the recognition of warning signs, contribute to obstetric peregrination, and negatively affect maternal and infant outcomes^(35)^. Thus, the strategic role of Fluvial Primary Health Units (*UBSF*) is reinforced in implementing continuous, culturally adapted educational actions aligned with primary care policies, in order to improve the quality of care offered to riverside populations^(11,12)^.

Educational activities carried out during prenatal care have a direct influence on the health and autonomy of pregnant women, especially adolescents experiencing their first pregnancy. National studies indicate that these practices occur in approximately 40% to 58% of consultations, although they remain insufficient, particularly regarding guidance on vaginal birth and postpartum care^(22)^.

In the present study, more than half of the pregnant women received information about the referral maternity hospital, an essential aspect for birth planning in hard to reach contexts. The absence or insufficiency of such guidance contributes to obstetric peregrination - that is, the forced displacement of pregnant women in search of care - a phenomenon more frequent in the North and Northeast regions, especially among women with lower educational attainment^(32-35)^. This gap reinforces the need to reorganize care pathways and strengthen continuous educational strategies to ensure equitable and humanized obstetric care for populations living in geographically isolated territories.

The results of this study reaffirm the strategic relevance of Primary Health Care in planning, coordinating, and monitoring prenatal care in hard to reach regions. Pregnant women also expressed satisfaction with prenatal care, highlighting the importance of the continuous follow up provided by the UBSF teams. However, 28% reported dissatisfaction related to waiting times and insufficient counseling, revealing areas for improvement. Even so, the monthly presence of the UBSF in riverside communities was highly valued, as it helps reduce geographic barriers and expand access to comprehensive care, ensuring a constitutional right historically limited in these communities^(11,12)^.

The findings of this study further reinforce the essential role of Primary Health Care in planning, coordinating, and monitoring prenatal care in remote regions. Strengthening multidisciplinary teams - especially those working in Fluvial Primary Health Units - is crucial to expanding the reach of health actions and ensuring continuity of gestational care. Recognizing the geographic, cultural, and social specificities of riverside populations enables the development of more sensitive and effective interventions, contributing to reducing inequalities in access and promoting equity in maternal health.

### Study limitations

Data collection was carried out during a specific and limited period, which may restrict the identification of seasonal variations that influence both access to prenatal services and pregnant women’s adherence to follow up. The small number of participants limits the generalization of the findings and comparisons with other groups of riverside and non riverside pregnant women. Furthermore, the unique environment - characterized by dense forest, extensive rivers, and heavy rainfall - posed logistical challenges for the research team, hindering the acquisition of scientific information

### Contributions to the field of Nursing

This study highlights the relevance of Nursing in providing prenatal care to pregnant women living in conditions of social and geographic vulnerability, supporting adherence to gestational follow up in remote areas. From a public health perspective, the findings reinforce the need to expand both the coverage and the quality of prenatal care, especially in hard to reach territories. In addition, the data may inform primary care policies, contributing to strengthening the structure of Fluvial Primary Health Units (UBSF) and promoting equity in care, with positive impacts on maternal and infant outcomes.

## CONCLUSIONS

Prenatal care in riverside communities of the Amazon met the criteria established by the Ministry of Health, with emphasis on the role of the itinerant team in promoting maternal and child well being. The Fluvial Primary Health Unit (UBSF) contributed to expanding coverage and reducing geographic barriers to access. However, challenges remain regarding continuity of care and adaptation to local conditions, reinforcing the need for equity and improvement in service delivery.

## Data Availability

The research data are available only upon request.
