# Supplementary material for: Quality of prenatal care in riverside communities of the Brazilian Amazon
Source: Rev Bras Enferm. 2026 Nov 6;79:e20250204. doi: 10.1590/0034-7167-2025-0204 (PMC13742975; doi:10.1590/0034-7167-2025-0204)
Supplement: Translation

# Translation: Qualidade da assistência pré-natal em comunidades ribeirinhas da Amazônia brasileira

**DOI:** 10.1590/0034-7167-2025-0204pt

**Authors:** Edson Silva do Nascimento, Anderson Lima Cordeiro da Silva, Bibiane Dias Miranda Parreira, Mônica Maria de Jesus Silva, Nayara Gonçalves Barbosa, Alexandre Faisal Cury, Flávia Gomes-Sponholz

**Affiliations:** I Universidade de São Paulo. Ribeirão Preto, São Paulo, Brasil; II Universidade Federal do Triângulo Mineiro. Uberaba, Minas Gerais, Brasil; III Universidade de São Paulo. São Paulo, São Paulo, Brasil

**Keywords:** Enfermagem, Qualidade da Assistência à Saúde, Atenção Primária à Saúde, Cuidado Pré-Natal, Serviços de Saúde Rural.

## Abstract

**Objetivos:**

descrever a qualidade da assistência pré-natal oferecida às gestantes ribeirinhas de Manaus, Amazonas.

**Métodos:**

estudo transversal realizado em dezembro de 2022 em uma Unidade Básica de Saúde Fluvial de Manaus/AM. Participaram 25 gestantes ribeirinhas, com idade ≥18 anos, residentes na área de abrangência, e com pelo menos uma consulta registrada. Os dados foram coletados por entrevistas e consulta aos registros durante expedição e analisados por estatística descritiva no SPSS v.20.0

**Resultados:**

todas realizaram pré-natal, metade não atingiu seis consultas. Foram ofertados exames, vacinas e procedimentos clínico-obstétricos, com maior regularidade na aferição da pressão arterial e ausculta fetal. Houve lacunas nas orientações sobre sinais de risco e maternidade de referência, embora a maioria tenha relatado satisfação com a assistência.

**Conclusões:**

apesar da cobertura e dos avanços promovidos pela UBSF na ampliação do acesso, persistem desafios para assegurar continuidade e equidade do cuidado em áreas remotas, ressaltando a necessidade de aperfeiçoamentos.

## INTRODUÇÃO

A atenção pré-natal é considerada pelo Ministério da Saúde (MS) um componente decisivo da linha de cuidado materno-infantil, pois contribui para uma gestação, parto e puerpério mais seguros, favorecendo o bem-estar da mulher e do recém-nascido^(1)^. Esse acompanhamento permite identificar precocemente situações de risco e fortalecer o vínculo entre usuárias e equipes da atenção básica^(2)^. Com a implantação do Programa de Humanização do Pré-Natal e Nascimento (2000) e da Rede Alyne (2024), ambas iniciativas do Ministério da Saúde, buscou-se aprimorar a estrutura, a organização e a expansão da assistência materno-infantil, ampliando o enfoque na equidade e nos direitos reprodutivos, e mantendo o pré-natal como eixo estruturante da rede de cuidados^(3)^.

O contexto brasileiro é marcado por ampla diversidade territorial, étnica e cultural^(4)^. Nesse cenário de desigualdades regionais, a Amazônia brasileira se destaca como um território onde as limitações estruturais, logísticas e organizacionais dos serviços de saúde se expressam de forma mais intensa, tornando-se um contexto emblemático para a análise da equidade na atenção pré-natal. Entre os grupos populacionais que vivem em condições desafiadoras de acesso aos serviços de saúde, destacam-se as comunidades ribeirinhas amazônicas, cujas formas de vida se organizam às margens dos rios e dependem da logística fluvial^(4)^. Nessas localidades, as distâncias geográficas, a escassez de transporte regular e as limitações estruturais dos serviços públicos constituem barreiras relevantes ao acompanhamento pré-natal contínuo e de qualidade^(5,6)^. Compreender as condições de vida e saúde dessas gestantes é fundamental para orientar ações de cuidado culturalmente sensíveis e estratégias de ampliação do acesso^(6,7)^.

Essas desigualdades estruturais e territoriais refletem-se de maneira concreta nos indicadores de atenção pré-natal em diferentes contextos da Amazônia, evidenciando como as limitações de acesso impactam populações que vivem em áreas remotas. Embora se refiram a populações indígenas, dados do Distrito Sanitário Especial Indígena de Manaus (DSEI/Manaus) oferecem subsídios importantes para compreender as limitações estruturais da atenção pré-natal em territórios amazônicos remotos, e que também afetam outras populações tradicionais, como as comunidades ribeirinhas. Em relatórios recentes do DSEI/Manaus, cerca de 52% das gestantes indígenas completaram o pré-natal com seis ou mais consultas^(8)^. Entre gestantes ribeirinhas, especificamente, um estudo realizado em comunidades do Médio Solimões com 28 mulheres apontou elevada proporção de primeira gestação na adolescência e alta frequência de partos domiciliares, refletindo padrões reprodutivos do contexto local^(9)^. Esses achados reforçam a relevância de investigar não apenas a cobertura, mas a qualidade da atenção pré-natal nas populações ribeirinhas amazônicas.

Para além da descrição dos indicadores de cobertura, estudos recentes abordam que a organização dos serviços de saúde e as condições territoriais desempenham papel central na conformação da atenção pré-natal em comunidades amazônicas^(5,6)^. A heterogeneidade territorial da Amazônia, caracterizada por extensas distâncias, dispersão populacional e dependência da logística fluvial, repercute diretamente na organização e no funcionamento dos serviços de saúde. Nesse contexto, a disponibilidade periódica das unidades de saúde fluviais, as limitações operacionais e a necessidade de adaptação do fluxo assistencial condicionam a oferta de consultas, exames e orientações, exigindo que os modelos assistenciais do SUS sejam estruturados de acordo com as especificidades socioeconômicas e epidemiológicas de cada localidade^(10)^.

Nesse sentido, a Política Nacional de Atenção Básica buscou ampliar a oferta de serviços e reorganizar o cuidado para atender às necessidades específicas de populações em áreas de difícil acesso. Como parte dessa estratégia, o Ministério da Saúde estabeleceu, por meio da Portaria n. 2.191 de 3 de agosto de 2010, as Equipes de Saúde da Família Fluviais e Ribeirinhas, com foco na atenção às comunidades da Amazônia Legal e do Pantanal Sul-Mato-Grossense^(11,12)^.

Evidências recentes indicam alta cobertura da atenção pré-natal no Brasil, com estimativas de cerca de 98,7% em municípios de pequeno e médio porte^(13)^. Entretanto, essa ampla cobertura não se traduz de forma homogênea em início oportuno do acompanhamento, número adequado de consultas e realização sistemática dos procedimentos recomendados, persistindo diferenças importantes entre regiões e contextos assistenciais^(14)^. Em áreas de difícil acesso, como determinadas localidades da Amazônia, essas assimetrias evidenciam limites dos modelos convencionais de organização da atenção básica, reforçando a necessidade de estratégias assistenciais adaptadas às especificidades territoriais e populacionais^(10)^.

Esse quadro não se restringe ao Brasil. Em outros países do Sul Global, como na região de Assam, Índia, mulheres relatam conhecimento limitado sobre direitos reprodutivos e enfrentam dificuldades de acesso aos serviços de planejamento familiar^(15)^. Esse paralelo contribui para ampliar a compreensão de que as barreiras enfrentadas por populações tradicionais em contextos remotos decorrem de condicionantes estruturais e territoriais compartilhados, não configurando como uma exclusividade do contexto amazônico ou brasileiro. Nesse sentido, obstáculos semelhantes, relacionados a barreiras geográficas, culturais e econômicas, têm sido descritos como fatores que condicionam práticas reprodutivas e limitam a autonomia das mulheres em diferentes contextos^(16)^.

A presente investigação se justifica pela necessidade de compreender como as condições geográficas e socioeconômicas influenciam o acesso e a continuidade da atenção pré-natal em regiões remotas da Amazônia. Tais fatores permanecem pouco explorados na literatura e configuram elementos centrais para o aprimoramento da equidade e da qualidade da assistência obstétrica oferecida às mulheres ribeirinhas.

### Relevância do estudo

Este artigo é produto da Dissertação de Mestrado intitulada “Análise da qualidade da assistência pré-natal em comunidades ribeirinhas da Amazônia brasileira”, defendida na Universidade de São Paulo^(17)^.

## OBJETIVOS

Descrever a qualidade da assistência pré-natal oferecida às gestantes ribeirinhas do município de Manaus, Amazonas.

## MÉTODOS

### Aspectos éticos

A pesquisa foi aprovada pelo Comitê de Ética em Pesquisa da Escola de Enfermagem de Ribeirão Preto da Universidade de São Paulo, em conformidade com a Resolução nº 466/2012 do Conselho Nacional de Saúde, que estabelece diretrizes para pesquisas envolvendo seres humanos.

### Desenho, período e local do estudo

Trata-se de um estudo descritivo, realizado entre 13 e 21 de dezembro de 2022, em uma Unidade Básica de Saúde Fluvial (UBSF) que percorre a calha do rio Amazonas, área rural no município de Manaus, Amazonas. O delineamento seguiu as recomendações do *Strengthening the Reporting of Observational Studies in Epidemiology* (STROBE).

A calha do rio Amazonas possui cobertura da Estratégia Saúde da Família por meio de uma UBSF vinculada à Secretaria Municipal de Saúde que, de modo itinerante, oferece assistência mensal às comunidades dispersas ao longo do percurso fluvial. Em toda a região existem seis polos de Unidades Básicas de Saúde Ribeirinhas, que abrangem 23 localidades - entre comunidades, igarapés, lagos e ramais - assistidas pela UBSF e por Agentes Comunitários de Saúde que permanecem nessas localidades, mesmo na ausência da embarcação^(18)^.

A região contava com cerca de 2.333 habitantes^(18)^ distribuídos ao longo do leito fluvial, em extensa área aquática distante do centro administrativo de Manaus. As atividades de subsistência incluem agricultura familiar (com destaque para a produção e comercialização da farinha de mandioca), pesca artesanal, extrativismo agropecuário e, eventualmente, a caça.

### População ou amostra, critérios de inclusão e exclusão

Participaram 25 gestantes acompanhadas pela UBSF durante o pré-natal, selecionadas por amostragem de conveniência, diante da impossibilidade de acesso prévio a informações que permitissem o cálculo amostral, considerando as limitações logísticas de uma expedição fluvial. A inclusão ocorreu conforme os critérios estabelecidos e a presença das gestantes nas consultas de pré-natal realizadas durante a permanência da UBSF nas comunidades. Reconhece-se que o uso da amostra por conveniência pode introduzir viés de seleção e restringir a representatividade dos resultados. Entretanto, essa estratégia mostrou-se viável frente às barreiras geográficas da região e permitiu descrever de modo consistente a realidade das mulheres atendidas.

Os critérios de inclusão foram idade igual ou superior a 18 anos, residência em comunidade ribeirinha da área de abrangência da UBSF, e registro de pelo menos uma consulta de pré-natal. Os critérios de exclusão abrangeram gestantes que interromperam o acompanhamento após a primeira consulta, que não compreendiam o idioma português ou que possuíam deficiência auditiva total ou parcial que impossibilitasse a entrevista. Durante a coleta, nenhuma gestante foi excluída, seja por interrupção do acompanhamento seja por limitações sensoriais. A coleta ocorreu em contexto de uma expedição fluvial, o que inviabilizou a busca ativa, restringindo a participação às gestantes atendidas presencialmente.

### Protocolo do estudo

Antes do início da coleta, foi realizada uma reunião preparatória no Distrito de Saúde Rural, com apresentação do pesquisador e do projeto à equipe da UBSF. O encontro também contou com a participação dos operadores da embarcação, com o propósito de compreender o funcionamento da unidade e organizar o fluxo de atendimento à população, favorecendo uma recepção acolhedora às participantes do estudo.

A coleta de dados foi realizada na própria embarcação, após a consulta de pré-natal, entre segunda-feira e domingo, das 8h às 12h e das 14h às 17h. As entrevistas foram conduzidas pelo pesquisador responsável, que explicou os objetivos, procedimentos e eventuais riscos do estudo. Após a leitura e assinatura do Termo de Consentimento Livre e Esclarecido, as entrevistas ocorreram de forma individual e privativa, mediante o uso de instrumento impresso. Não houve recusas nem perdas de informações.

As variáveis para caracterizar o perfil sociodemográfico e econômico incluíram idade, raça/cor, situação conjugal, escolaridade, religião, estado civil, ocupação, renda individual e familiar, provedor da família e número de moradores do domicílio.

A avaliação da qualidade da assistência pré-natal contemplou realização do último pré-natal, procedimentos clínico-obstétricos, medidas preventivas, orientações de saúde recebidas e satisfação com o acompanhamento.

O instrumento de caracterização sociodemográfica foi elaborado com base em literatura científica nacional e internacional sobre populações em contextos vulneráveis.

O instrumento empregado para avaliar a qualidade da assistência foi adaptado a partir do estudo conduzido em Nampula, Moçambique^(19)^, selecionado em razão de sua abrangência e aplicabilidade em contextos de vulnerabilidade e difícil acesso, como o das comunidades ribeirinhas amazônicas, permitindo a mensuração direta da qualidade da assistência recebida.

### Análise dos resultados e estatística

Os dados foram duplamente digitados em planilha eletrônica no *Microsoft Excel*, para verificação de consistência, e analisados por meio do software IBM SPSS Statistics, versão 20.0, utilizando estatística descritiva.

As variáveis contínuas foram apresentadas como médias e desvios-padrão, e as variáveis categóricas como frequências absolutas e relativas (n, %).

Ressalta-se que, por se tratar de um delineamento transversal e de amostra reduzida obtida por conveniência, não foi possível estabelecer relações de causalidade. As dificuldades de acesso típicas do contexto fluvial configuram limitações adicionais para a generalização do estudo

## RESULTADOS

Participaram do estudo 25 gestantes residentes em comunidades situadas ao longo da calha do rio Amazonas, acompanhadas durante o pré-natal em uma Unidade Básica de Saúde Fluvial (UBSF). A maioria era jovem (64% entre 20 e 29 anos), vivia em união estável (88%), apresentava baixa escolaridade e condição socioeconômica vulnerável, com dependência de benefícios de transferência de renda como o Bolsa Família (68%), além de vínculo com atividades agrícolas locais (Tabela 1). Esse perfil revela a sobreposição entre vulnerabilidades sociais e limitações estruturais, especialmente no acesso e na renda, que influenciam as trajetórias de cuidado das gestantes ribeirinhas.

**Tabela 1:** Características socioeconômicas das gestantes residentes na calha do rio Amazonas em acompanhamento pré-natal na Unidade Básica de Saúde Fluvial, Manaus, Amazonas, Brasil, 2023

Variáveis	n	%
**Faixa etária (anos completos)**		
18 -| 19	4	16
20 -| 29	16	64
30 -| 39	4	16
40 -| 49	1	4
**Cor autorreferida**		
Parda	21	84
Amarela	3	12
Preta	1	4
**Escolaridade formal**		
Ensino Fundamental Incompleto	7	28
Ensino Médio Completo	7	28
Ensino Fundamental Completo	5	20
Ensino Médio Incompleto	3	12
Ensino Superior Completo	2	8
Ensino Superior Incompleto	1	4
**Religião**		
Católica	10	40
Evangélica	7	28
Crê em Deus, mas não tem religião	2	8
Outras	6	24
**Estado civil**		
Casada	22	88
Solteira	3	12
**Ocupação**		
Do lar	19	76
Mercado formal	2	8
Desempregada	2	8
Mercado informal	1	4
Estudante	1	4
**Fonte de renda**		
Bolsa Família	17	68
Não possui renda	3	12
Mercado Formal	2	8
Auxílio Estadual	1	4
Mercado Informal	1	4
Pensão	1	4
**Renda individual (em salário mínimo)**		
≤ 1	22	88
1-| 2	1	4
Sem renda individual	2	8
**Renda familiar (em salário mínimo)**		
≤ 1	13	52
1-| 2	11	44
Sem renda familiar	1	4
**Provedor da família**		
Companheiro	8	32
Ambos (participante e companheiro)	10	40
Participante	3	12
Pais	3	12
Outros	1	4
**Número de pessoas residentes no domicílio**		
2	4	16
3	8	32
4	5	20
Mais de 5	8	32
**Total**	25	100,0

Todas as participantes realizaram acompanhamento pré-natal. Aproximadamente metade não completou seis consultas, principalmente em razão das dificuldades de transporte e deslocamento características do contexto ribeirinho, enquanto a outra metade atingiu o número mínimo recomendado, favorecida pelo apoio familiar, pela disponibilidade de meios de transporte e pela maior proximidade da UBSF (Tabela 2).

**Tabela 2:** Características da realização do último pré-natal das gestantes residentes na calha do rio Amazonas em acompanhamento pré-natal na Unidade Básica de Saúde Fluvial, Manaus, Amazonas, Brasil, 2023

Variáveis	Sim		Não	
	n	%	n	%
**IG de início do pré-natal (em semanas)**				
1 -| 4			9	36
5 -| 8			10	40
9 -| 12			6	24
**Motivos de ter realizado < 6 consultas**				
Dificuldades relacionadas ao trabalho/escola	3	12	22	88
Não sabia que estava grávida	3	12	22	88
Não aceitava a gestação	3	12	22	88
Achou a barriga pequena	2	8	23	92
Não tinha quem a acompanhasse	2	8	22	92
Atendimento demorado	1	4	24	96
Não achava importante iniciar o PN cedo	1	4	24	96
Não tinha com quem deixar os filhos	1	4	24	96
UBSF de saúde fica distante	1	4	24	96
**Motivos de ter realizado > 6 consultas**				
Apoio familiar e do parceiro	19	76	6	24
Reconhece a importância do pré-natal	19	76	6	24
UBSF de saúde próximo de casa	19	76	6	24
Acesso ao transporte	18	72	7	28
Desejo de cuidar melhor da gestação	18	72	7	28
Profissionais atendem bem	16	76	6	24
**Número total de consultas de pré-natal realizadas (até o dia da entrevista)**				
1-| 3			6	24
4-| 5			6	24
≥ 6			13	52
**Total**			**25**	**100,0**

Observou-se oferta consistente de exames básicos, entre eles a ultrassonografia (88%), além de vacinas e procedimentos clínico-obstétricos, com destaque para a aferição da pressão arterial (60%) e a ausculta fetal (60%). A cobertura vacinal apresentou níveis satisfatórios para hepatite B (84%), tétano (80%), influenza (88%), embora a adesão à vacina contra a COVID-19 tenha sido inferior (48%), considerando as três doses recomendadas pelo Ministério da Saúde (Tabela 3). Esses achados evidenciam o papel da UBSF como estratégia itinerante de cuidado, capaz de garantir a oferta de serviços essenciais mesmo em regiões de difícil acesso.

**Tabela 3:** Características das gestantes residentes na calha do rio Amazonas em acompanhamento pré-natal na Unidade Básica de Saúde Fluvial, segundo a realização de procedimentos clínico-obstétricos e medidas preventivas realizadas durante o pré-natal, Manaus, Amazonas, Brasil, 2023

Variáveis	Nenhuma		1		2		3		≥4	
	n	%	n	%	n	%	n	%	n	%
Número de avaliações da pressão arterial	2	8	2	8	1	4	5	20	15	60
Número de avaliações do peso	2	8	1	4	2	8	6	24	14	56
Número de ofertas de sulfato ferroso	-	-	7	28	5	20	4	16	9	36
Número de medições da altura uterina	1	4	2	8	1	4	7	28	12	48
Número de auscultas dos BCF	2	8	1	4	2	8	3	12	15	60
Vacina da Hepatite B	1	4	3	12	-	-	21	84	-	-
Vacina Dtpa	5	20	20	80	-	-	-	-	-	-
Vacina Antitetânica	1	4	1	16	-	-	20	80	-	-
Vacina contra Influenza	3	12	22	88	-	-	-	-	-	-
Vacina contra Covid-19	1	4	1	4	7	28	12	48	4	16

Os registros referentes aos sinais de risco gestacional foram pouco frequentes (8 -16%), o que indica uma lacuna na qualidade da atenção pré-natal ofertada às gestantes.

As orientações mais frequentes abordaram alimentação saudável (88%), cuidados pós-parto (76%) e planejamento reprodutivo (72%), enquanto sinais de risco gestacional foram pouco tratados. Essa lacuna reflete o impacto das restrições de tempo e da rotatividade das equipes durante as expedições fluviais.

A satisfação com o atendimento foi predominante (96%), destacando-se a cordialidade da equipe e a valorização da presença da UBSF nas comunidades. Essa percepção positiva evidencia como a oferta regular de serviços em expedições fluviais contribui para reduzir barreiras geográficas e fortalecer o vínculo entre a comunidade e o serviço de saúde.

## DISCUSSÃO

O grupo de gestantes investigado era predominantemente jovem, com baixa escolaridade e inserido em contextos de vulnerabilidade socioeconômica. Essa condição se manifesta tanto nos meios de subsistência quanto nas suas dificuldades de acesso aos serviços de saúde, coincidindo com achados do estudo nacional sobre a adequação do cuidado pré-natal^(1)^ e com resultados obtidos em comunidades amazônicas, como a vila Alvarães^(20)^. Nesse contexto, a Política Nacional de Saúde Integral das Populações do Campo, da Floresta e das Águas desempenha papel estratégico na ampliação do acesso ao pré-natal entre populações rurais e tradicionais^(21,22)^.

A renda das participantes provinha majoritariamente de programas de transferência de renda, especialmente o auxílio social, frequentemente utilizado para cobrir necessidades básicas. A insuficiência de recursos financeiros é recorrente nas comunidades ribeirinhas amazônicas^(20)^ e, em determinados períodos, foi compensada pela agricultura de subsistência e pela venda local de produtos como a mandioca^(20)^. Essas condições econômicas restringem o deslocamento e a continuidade do acompanhamento pré-natal, reforçando a importância de políticas de proteção social como estratégia de equidade no acesso à saúde.

As desigualdades socioeconômicas entre as regiões brasileiras influenciam o comportamento reprodutivo, especialmente em contextos de maior vulnerabilidade, onde se observa com maior frequência a gravidez precoce e a multiparidade. Esse quadro agrava-se por barreiras geográficas e limitações de acesso a serviços especializados^(23)^. Nas regiões Norte e Nordeste, o início tardio e a menor cobertura do pré-natal entre mulheres com baixa escolaridade estão mais associados a barreiras territoriais e estruturais do que à falta de informação^(23)^, evidenciando a necessidade de estratégias que considerem as especificidades locais e assegurem o início oportuno do acompanhamento.

A qualidade do cuidado pré-natal relaciona-se ao início precoce do acompanhamento - preferencialmente entre o primeiro e o terceiro mês de gestação, com a realização adequada de consultas, exames e orientações consistentes sobre a maternidade de referência, fatores essenciais para aprimorar os desfechos perinatais^(24)^.

Pesquisas conduzidas em distintas regiões do Brasil, incluindo o Norte, Nordeste, Sudeste e Sul apontam que, de modo geral, o número médio de consultas e início do pré-natal ocorrem dentro do período recomendado^(24,25)^. Esses resultados demonstram capacidade da atenção básica para assegurar atendimento adequado, inclusive em localidades remotas. De modo semelhante, uma investigação realizada na região amazônica identificou elevada proporção de início precoce do acompanhamento pré-natal^(5)^, enquanto em Altamira/PA, observou-se início tardio e menor número de consultas^(26)^, evidenciando diferenças entre os estados amazônicos em relação às recomendações ministeriais.

Em contextos internacionais com populações dispersas e recursos limitados, observa-se um panorama diverso, como a alta cobertura em Vanuatu, país insular do Pacífico Sul^(27)^, e baixa frequência de consultas em áreas rurais da Zâmbia^(16)^.

Na região estudada, o acompanhamento pré-natal é realizado mensalmente por equipe multiprofissional composta por médicos e enfermeiros, organização que não segue a alternância de atendimentos preconizada nos protocolos nacionais^(3)^. Essa configuração assistencial se aproxima do observado em áreas rurais de Manaus (AM) e de Lagarto (SE)^(28)^, onde a limitação de profissionais e as longas distâncias condicionam o modelo assistencial. Nos estados do Norte e Nordeste o acompanhamento pré-natal costuma ser conduzido principalmente por enfermeiros, dada a escassez de médicos nos municípios do interior^(29)^. Em cenários internacionais semelhantes, como nas ilhas do Estreito de Torres na Austrália, modelos de atenção liderados por enfermeiros mostraram-se eficazes no início oportuno do pré-natal e na ampliação do número médio de consultas^(30)^.

Verificou-se elevada adesão aos exames laboratoriais na primeira consulta de pré-natal, indicando o cumprimento dos protocolos básicos de rastreamento. A equipe da UBSF ampliou essa rotina por meio da solicitação de exames complementares, incluindo sorologias e investigação de plasmódio, adequando o cuidado às condições endêmicas da região amazônica. Esse desempenho contrasta com o observado em outras regiões urbanas ou não remotas do país, onde a realização desses exames tende a ser menos frequente^(31)^.

Embora a malária seja endêmica na região, nenhuma gestante referiu diagnóstico positivo durante o acompanhamento, conforme resultados do exame de gota espessa, procedimento preconizado pela Rede Cegonha no pré-natal nos estados da Amazônia Legal. Pesquisas conduzidas em Manaus, Coari e na Colômbia identificam maior vulnerabilidade das gestantes à infecção. Em Coari, o risco mostrou-se até cinco vezes superior ao observado entre mulheres que não estavam grávidas^(32)^. Esses achados reforçam a necessidade de manter a vigilância ativa e o rastreamento sistemático da malária durante o pré-natal, a fim de reduzir riscos materno e fetais.

Verificou-se que a maioria das gestantes realizou os principais procedimentos clínicos e preventivos do pré-natal, em conformidade com as diretrizes do Ministério da Saúde^(2,4)^. Esses resultados são compatíveis com estudos desenvolvidos em áreas remotas do Norte do Brasil, que ressaltam a relevância da continuidade dos cuidados gestacionais para a detecção precoce de riscos e agravos^(22,31)^.

No presente estudo, aferição de pressão arterial, peso e estatura foram frequentemente realizados pelos Agentes Comunitários de Saúde, como estratégia de otimização do tempo de atendimento durante a permanência da UBSF nas comunidades^(33)^. A cobertura vacinal apresentou indicadores satisfatórios, superiores aos descritos em estudo realizado em uma maternidade do Nordeste^(34)^. Em contrapartida, pesquisa conduzida em Nampula, Moçambique, aponta menor cobertura^(19)^ do que a observada no Brasil^(30)^, evidenciando disparidades entre contextos remotos e a necessidade de ampliar a cobertura vacinal nacional mesmo diante do acesso gratuito garantido pelo SUS.

Foram identificadas fragilidades e lacunas nas ações educativas do pré-natal, sobretudo na promoção do parto vaginal, conforme também descrito em estudos realizados no Brasil^(13)^ e em Moçambique^(19)^. A sobrecarga das equipes é reconhecida como um fator que limita a oferta de orientações e compromete a preparação das gestantes ao longo do ciclo gravídico-puerperal^(31)^. Essas fragilidades podem atrasar o reconhecimento de sinais de risco, favorecer a peregrinação obstétrica e impactar negativamente os desfechos materno-infantis^(35)^. Assim, reforça-se o papel estratégico das UBSF na implementação de ações educativas contínuas, culturalmente adaptadas e alinhadas às políticas de atenção básica, de modo a qualificar o cuidado oferecido às populações ribeirinhas^(11,12)^.

As atividades educativas realizadas durante o pré-natal exercem influência direta sobre a saúde e a autonomia das gestantes, especialmente das adolescentes que vivenciam sua primeira gestação. Pesquisas nacionais indicam que essas práticas são realizadas em aproximadamente 40% a 58% dos atendimentos, embora permaneçam insuficientes sobretudo no que se referem às orientações sobre o parto vaginal e cuidados no puerpério^(22)^.

No presente estudo, observou-se que mais da metade das gestantes recebeu informações sobre a maternidade de referência, aspecto essencial para o planejamento do parto em contextos de difícil acesso. A ausência ou insuficiência dessas orientações contribui para a ocorrência de peregrinação obstétrica, isto é, o deslocamento forçado de gestantes em busca de assistência, fenômeno mais frequente nas regiões Norte e Nordeste, sobretudo entre mulheres com menor escolaridade^(32-35)^. Essa fragilidade reforça a necessidade de reorganização dos fluxos assistenciais e fortalecimento das estratégias educativas contínuas de modo a assegurar um cuidado obstétrico equitativo e humanizado para populações que vivem em territórios de difícil acesso.

Os resultados deste estudo reafirmam a relevância estratégica da Atenção Básica no planejamento, coordenação e acompanhamento do cuidado pré-natal em regiões de difícil acesso. As gestantes também demonstraram satisfação com a assistência pré-natal, ressaltando a importância do acompanhamento contínuo realizado pelas UBSF. No entanto, 28% relataram insatisfação em relação ao tempo de espera e à escassez de orientações, evidenciando pontos de melhoria. Ainda assim, a presença mensal da UBSF nas comunidades ribeirinhas foi valorizada, pois contribui para reduzir barreiras geográficas e ampliar o acesso ao cuidado integral, assegurando um direito constitucional historicamente limitado nessas comunidades^(11,12)^.

Os resultados deste estudo reafirmam a relevância estratégica da Atenção Básica no planejamento, coordenação e acompanhamento do cuidado pré-natal em regiões de difícil acesso. O fortalecimento das equipes multiprofissionais - especialmente aquelas que atuam em Unidades Básicas de Saúde Fluviais - é essencial para ampliar o alcance das ações e garantir continuidade ao cuidado gestacional. Reconhecer as particularidades geográficas, culturais e sociais das populações ribeirinhas permite desenvolver intervenções mais sensíveis e efetivas, contribuindo para a redução das desigualdades no acesso e para a promoção da equidade em saúde materna.

### Limitações do estudo

A coleta de dados foi realizada em um período específico e delimitado, o que pode limitar a identificação de variações sazonais que influenciam tanto o acesso aos serviços de pré-natal quanto a adesão das gestantes ao acompanhamento. O número reduzido de participantes restringe a generalização dos achados e as comparações com outros grupos de gestantes ribeirinhas e não ribeirinhas. Ademais, o ambiente singular caracterizado por densa floresta, extensos rios e chuvas intensas impôs desafios logísticos à equipe de pesquisa, dificultando a obtenção de informações científicas.

### Contribuições para a Área

Este estudo evidencia a relevância da Enfermagem na atenção pré-natal de gestantes em situação de vulnerabilidade social e geográfica, favorecendo a adesão ao acompanhamento gestacional em áreas remotas. Em termos de saúde pública, os achados reforçam a necessidade de ampliar tanto a cobertura quanto a qualidade da atenção pré-natal, especialmente em territórios de difícil acesso. Além disso, os dados podem subsidiar políticas de atenção primária, contribuindo para o fortalecimento da estrutura das UBSF e para a promoção da equidade no cuidado, com impactos positivos sobre os desfechos materno-infantis.

## CONCLUSÕES

A assistência pré-natal nas comunidades ribeirinhas do Amazonas atendeu aos critérios do Ministério da Saúde, com destaque para a atuação da equipe itinerante na promoção do bem-estar materno-infantil. A UBSF contribuiu para ampliar a cobertura e reduzir barreiras geográficas de acesso. Contudo, persistem desafios relacionados à continuidade do cuidado e à adaptação às condições locais, reforçando a necessidade de equidade e aprimoramento da assistência.

## Data Availability

Os dados de pesquisa só estão disponíveis mediante solicitação.
